# Quantitative Backscattered Electron Imaging of Bone Using a Thermionic or a Field Emission Electron Source

**DOI:** 10.1007/s00223-021-00832-5

**Published:** 2021-04-10

**Authors:** Markus A. Hartmann, Stéphane Blouin, Barbara M. Misof, Nadja Fratzl-Zelman, Paul Roschger, Andrea Berzlanovich, Gerlinde M. Gruber, Peter C. Brugger, Jochen Zwerina, Peter Fratzl

**Affiliations:** 1 Ludwig Boltzmann Institute of Osteology at Hanusch Hospital of OEGK and AUVA Trauma Centre Meidling, 1st Medical Department Hanusch Hospital, Heinrich Collin Strasse 30, 1140 Vienna, Austria; 2 Unit of Forensic Gerontology, Center of Forensic Science, Medical University of Vienna, Sensengasse 2, 1090 Vienna, Austria; 3 Department of Anatomy and Biomechanics, Karl Landsteiner University of Health Sciences, Dr.-Karl-Dorrek-Straße 30, 3500 Krems, Austria; 4 Center for Anatomy and Cell Biology, Department of Anatomy, Medical University of Vienna, Währingerstrasse 13, 1090 Vienna, Austria; 5 Department of Biomaterials, Max-Planck-Institute of Colloids and Interfaces, Am Mühlenberg 1, 14476 Potsdam, Germany

**Keywords:** Quantitative backscattered electron imaging, Bone mineralization density distribution, Adult human bone

## Abstract

Quantitative backscattered electron imaging is an established method to map mineral content distributions in bone and to determine the bone mineralization density distribution (BMDD). The method we applied was initially validated for a scanning electron microscope (SEM) equipped with a tungsten hairpin cathode (thermionic electron emission) under strongly defined settings of SEM parameters. For several reasons, it would be interesting to migrate the technique to a SEM with a field emission electron source (FE-SEM), which, however, would require to work with different SEM parameter settings as have been validated for DSM 962. The FE-SEM has a much better spatial resolution based on an electron source size in the order of several 100 nanometers, corresponding to an about $10^5$ to $10^6$ times smaller source area compared to thermionic sources. In the present work, we compare BMDD between these two types of instruments in order to further validate the methodology. We show that a transition to higher pixel resolution (1.76, 0.88, and 0.57 μm) results in shifts of the BMDD peak and BMDD width to higher values. Further the inter-device reproducibility of the mean calcium content shows a difference of up to 1 wt% Ca, while the technical variance of each device can be reduced to $\pm 0.17$ wt% Ca. Bearing in mind that shifts in calcium levels due to diseases, e.g., high turnover osteoporosis, are often in the range of 1 wt% Ca, both the bone samples of the patients as well as the control samples have to be measured on the same SEM device. Therefore, we also constructed new reference BMDD curves for adults to be used for FE-SEM data comparison.

## Introduction

From a material point of view, bone is a composite material of a soft and tough organic collagen matrix reinforced with stiff and brittle inorganic hydroxylapatite nano-crystals [1, 2]. During an individual’s life span, bone forming and resorbing cells (osteoblasts and osteoclasts, respectively) steadily remodel bone, thus, allowing for mechanical adaptation and repairing material fatigue event like cracks. Osteoblasts lay down new bone in the form of collagenous bone matrix (osteoid) that subsequently mineralizes. This mineralization process can be roughly separated in two phases. During the phase of primary mineralization, the newly formed bone achieves up to $70\%$ mineral content in few days, whereas it takes up to months and even years to complete mineralization in the subsequent phase of secondary mineralization [3–8]. This interplay of bone formation and resorption leads to a specific pattern of bone packets of different age and, thus, of different mineralization contents. A quantitative assessment of bone mineralization is important as many metabolic diseases, and medication may affect mineralization. Examples include (high and low bone turnover) osteoporosis [9–12], osteogenesis imperfecta [13–15], melorheostosis [16, 17], hypophosphatemia [18], hypophosphatasia [19, 20] as well as bisphosphonate [21–23] or teriparatide [24] treatment.

The clinical gold standard for characterization of the bone status of a patient is measuring the bone mineral density (BMD) via dual-energy X-ray absorptiometry (DXA). One of the major drawbacks of this technique is that the result of a DXA measurement is a 2-dimensional projection obtained from a radiography through the whole body. Thus, it is impossible to disentangle the effect of bone volume and matrix mineralization. In other words, DXA does not allow to discriminate if a larger absorption contrast stems from a larger amount of bone material in the beam path or from a higher mineralization of the bone matrix [25]. Another clinical sophisticated method is high-resolution peripheral quantitative computed tomography (HR-pQCT) [26, 27]. While it allows to measure 3-dimensional bone micro-architecture, the use of a polychromatic X-ray beam giving rise to beam hardening and its voxel resolution of approximately 80 μm limit the accuracy of the obtained volumetric BMD. The method of quantitative backscattered electron imaging (qBEI) has the potential to overcome these limitations. In contrast to x-rays used in DXA and HR-pQCT measurements, backscattered electrons provide information of mineralization density from a sample depth of only 1–2 micrometers when working with beam electron energies of 20 keV [28]. This means that instead of a 2D projection of a 3D measurement as is obtained in DXA, a qBEI analysis gives truly 2D information on bone matrix mineralization at the sample surface devoid of effects stemming from bone volume. Thus, the frequency histogram of degree in mineralization of the sample, the so-called bone mineralization density distribution (BMDD) can be obtained directly from the backscattered electron images [29]. The method was first suggested by Alan Boyde [30] and further refined by Paul Roschger who used it extensively [31, 32]. Other groups throughout the world have also used it to measure bone mineral content in pathological situations [33–36]. Recently, evaluation protocols were developed to also assess osteocyte lacunae number, size, and shape from qBE images [15, 18, 37, 38]. However, it has to be emphasized that this method—in contrast to DXA and HR-pQCT—requires a bone biopsy sample.

**Fig. 1:**
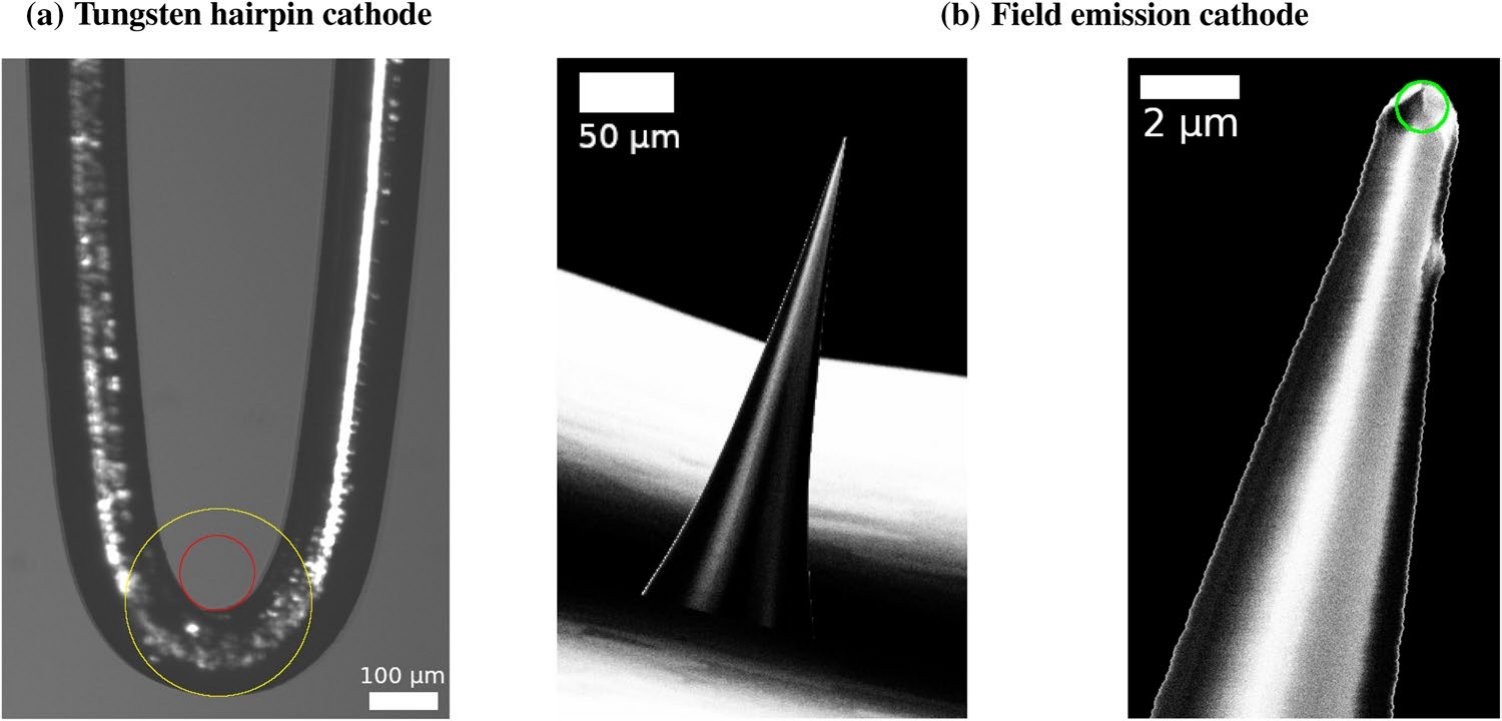
Representative images of the two types of cathodes discussed in the text. **a** Light microscopy image of a typical tungsten hairpin cathode used in the DSM 962. The red ($r=55 \ \mu \text {m}$) and yellow ($r=136 \ \mu \text {m}$) circles give an estimate of the source radius. **b** SEM image of the tip of a field emission cathode used in the SEM SUPRA 40 device. The green circle with $r=512 \ \text {nm}$ indicates the tip radius

The increasing popularity of the qBEI method for bone mineralization measurement makes it important to understand how results from different groups and devices with different electron sources can be compared. With respect to the electron source, existing SEMs can be classified in two groups: thermionic and field emission electron guns. The physical principle of thermionic electron guns is to heat the material to high temperatures to allow some electrons to overcome the work-function energy barrier and escape into vacuum via thermal excitation. As this type of cathodes relies on thermal excitation and, thus, has to be operated at elevated temperatures, the resulting current density and its life time are generally low. Typical thermionic electron sources are made from tungsten or Lanthanum Hexaboride (LaB$_6$). The second material has a lower working-function energy barrier compared to the first and, thus, can be operated at lower temperatures yielding a higher current density. In contrast to thermionic excitation, a field emission cathode uses the physical principle of electric field amplification close to sharp tips. A field emission cathode is a metal wire ending with a sharp tip with a radius of several $100 \ \text {nm}$. If this cathode is held at constant (negative) voltage, the electric field close to the tip becomes so large ($>10^9 \ \text {V/m}$) that the working energy barrier is reduced and electrons can escape into vacuum. The current density is approximately a factor $10^5$ larger compared to thermionic sources. Furthermore, the small source size of a field emission cathode results also in a small beam divergence and, thus, higher brightness (current per solid angle) compared to a thermionic source. As a field emission cathode can be operated at lower temperatures, its life time is considerably enhanced compared to a thermionic cathode (see also Fig. 1 for images of typical examples of the cathodes).

The qBEI method relies on the fact that in a scanning electron microscope (SEM), the number of backscattered electrons (BEs) is dependent on the local atomic number *Z* of the specimen. For low atomic numbers ($Z \lesssim 20$), this dependence is approximately linear [28]. The mean *Z* values of bone with a varying fraction of organic matrix ($Z \approxeq 6$) and hydroxylapatite ($Z=14.06$) lie well in this range, provided that in addition to the normal composition of bone, no significant amounts of other (heavier) elements are present. This allows to deduce the local mineral content in bone from the measured backscattered electron yield when an electron beam is scanned over a bone surface in a SEM that was calibrated by reference standards with known *Z* numbers prior to measurements [29].

In principle, the linear dependency of backscattered electron yield at low atomic numbers is actually only fulfilled if the sample material is homogeneous down to the atomic scale (randomly dispersed atoms) within the interaction volume, i.e., the sampling volume of BEs generated by the electron beam impinging the sample surface. This condition is well fulfilled for pure crystals provided that the strongly ordered arrangement of atoms on the sample surface is destroyed by proper mechanical polishing. Otherwise the BE yield would be additionally influenced by crystal orientation. In contrast, for bone, which is a nano-composite of organic molecules (mainly collagen) and nano-sized hydroxylapatite particles, the conditions of homogeneity within the sampling volume of BE are likely less strongly fulfilled. The dominant structural motif the bone material is built of, is the mineralized collagenous fibril of 50 to 200 nm in diameter impregnated by 4-nm-thick plate-like crystals. These mineralized fibrils compose a lamellar structure with a period of about 5 μm further forming the bone packets (basic structural units, BSUs). In consequence, not only the actual mineral content of the fibril, but also the amount of fibril material that is effectively sampled per pixel during scanning might influence the BE intensity. Thus, instrumental parameters like beam size, beam divergence, beam electron intensity as well as spatial pixel resolution will potentially contribute to a BE contrast additionally to that of atomic number contrast.

Nevertheless, the size and specificity of this effect are hitherto unexplored. This gap shall be closed in the current paper. We compare qBEI results from two different SEMs equipped with different electron sources. The first is a ZEISS DSM 962 with a tungsten hairpin cathode and the second a ZEISS field emission SEM SUPRA 40 (see Fig. 1 for images of the used cathodes). The former was used to validate the qBEI method and to establish reference BMDDs from healthy adults [32, 39]. Having these two different microscopes at hand and considering the issues discussed above, the question arises if measurements on the two devices are (quantitatively) comparable. In particular, this answers the question if measurements on the field emission SEM can be compared to the reference BMDDs obtained earlier with the DSM 962. The broader impact of this research question is to answer, how results from different research groups obtained with SEMs with different electron sources and operated with different experimental settings (e.g., nominal magnification), can be compared.

## Material and Methods

### Samples

The new reference curve on the SEM SUPRA 40 was decided to include only samples from the iliac crest from healthy individuals. Transiliac bone biopsy samples are by far the most common site for histopathological examination and diagnosis. Furthermore, using transiliac bone samples only allows to determine also a cortical reference BMDD (in contrast to the trabecular BMDD, the cortical BMDD is not site independent). For measurement of the adult reference BMDD iliac crest bone biopsy, samples from *N* = 25 individuals (7 males and 18 females) are chosen for the analysis. 13 of these samples had already been included in the previously published reference obtained with the DSM 962 (Note, that this reference included specimens also from other sites than the iliac crest) [39]. The remaining 12 specimens are obtained from autopsy samples of individuals with no known bone disease. All ethical requirements were approved by the Ethics Commission of the Medical University of Vienna (EK Nr. 1757/2013). The age range of all 25 samples is from 37 to 95 years (mean $68.7 \pm 19$ years). Each biopsy sample is partitioned in a cortical (comprising two cortices) and a trabecular region. All three regions are evaluated separately. The arithmetic mean of the two cortical BMDD curves of each sample defines the cortical sample’s BMDD [40]. The 25 trabecular and cortical BMDD curves are averaged to give the reference curve. The reference BMDD parameters represent the arithmetic mean of the reference population.

### Sample Preparation

Sample preparation is described in detail elsewhere [32, 39] and is only shortly recapitulated here. Native bone samples are immersed and stored in $70\%$ ethanol. The embedding process is started by an alcohol and acetone series to dehydrate and degrease the samples. Then the sample is embedded in poly-methylmethacrylate (PMMA). The hardening process takes several days at elevated temperatures of about 40 °C. When the hardening process is finished, the samples are trimmed and cut by a diamond saw (Buehler Isomet 1000) in a defined orientation for measurements. Then the surface is ground and polished (Logitech PM5). Finally, the sample is carbon coated (AGAR SEM carbon coater) to achieve a conducting surface. Together with the bone sample, an aluminum sample of high purity (99.9999%, MAC Consultants UK) is also carbon coated producing a carbon coating of similar thickness on the aluminum sample as on the bone sample. In the following, this is called the *sample standard* (in contrast to the *main standard* defined below). It is used to quantify the effect of the carbon deposition on the qBEI measurement.

### Instrumentation

The data presented in this work have been obtained through measurements with two scanning electron microscopes equipped with different electron sources. The first is a Zeiss DSM 962 with a tungsten hairpin cathode, the second a Zeiss field emission SEM SUPRA 40 equipped with a Schottky field emission electron gun with zirconium envelope. Figure 1 shows images of the two cathodes used. Note the difference in tip radius for both sources that leads to differences in beam characteristics like diameter and divergence. In both devices, the backscattered detector is a 4-quadrant solid-state detector but of different size and fabrication. With both devices, measurements were performed at 20 kV. The working distance was 15 mm for the DSM 962 and 10 mm for the SEM SUPRA 40, respectively. The probe current (measured as the specimen current of a faraday cup) was 110 pA for the DSM 962 and lay in the range of 280–320 pA for the SUPRA 40. These settings and a scanning speed of 90 s per image resulted in approximately the same electron exposure (electrons/pixel) of the sample for both devices.

To perform compositional analysis of samples using quantitative Energy Dispersive X-Ray spectroscopy (EDX), the SEM SUPRA 40 is equipped with an EDS Silicon Drift detector (X-Max, Oxford Instrument, UK). The analysis of EDX spectra is done using Oxford INCA software.

### Calibration

**Fig. 2:**
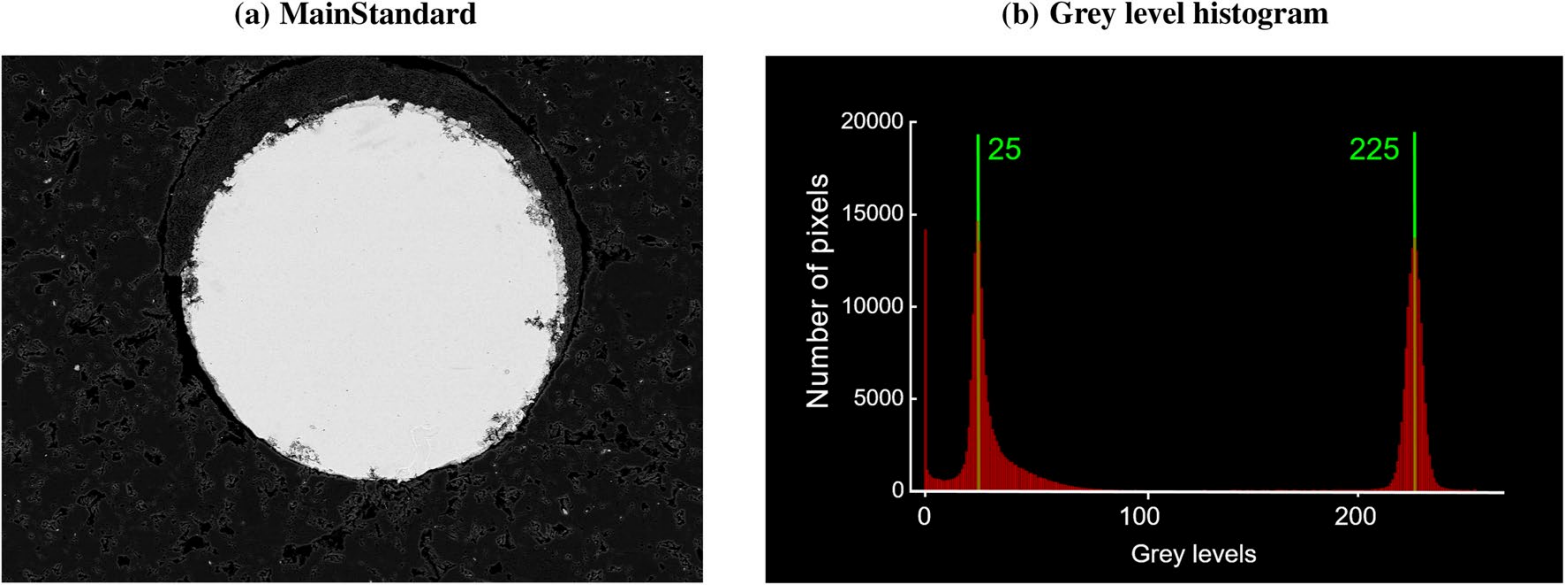
Calibration procedure for quantitative measurements. **a** The main standard that is used for calibration. The light area corresponds to aluminum, the dark ones to carbon. **b** Corresponding gray-level histogram. Brightness and contrast of the detector are adjusted that the gray levels corresponding to carbon and aluminum are centered at 25 and 225, respectively

The calibration procedure of the SEM includes two steps: (i) Since the BE signal intensity is highly sensitive to the working distance (WD) (i.e., the distance of the sample surface to the end of the SEM column and, thus, to the BE detector surface), the main standard (see below) and bone sample surface have to be adjusted for the exactly same WD. This is achieved by moving the samples mechanically into the fixed focal plane of electron beam. (ii) The backscattered signal is calibrated using two reference materials of known atomic number. We are using a dual-element reference sample of high purity containing aluminum (*Z* = 13) in the center surrounded by carbon (*Z* = 6) called the *main standard*. The calibration routine consists in tuning the brightness and contrast of the detector in order to obtain a gray level of $25\pm 1$ for carbon and $225\pm 1$ for aluminum. The gray value histogram from an image showing the main standard develops two distinct peaks. Brightness and contrast settings are used to shift both peaks simultaneously or to change the distance between them, respectively (see also Fig. 2).

### Carbon Coating Correction (for SEM SUPRA 40 Only)

When contrast and brightness values are set for the main standard, the carbon-coated aluminum sample standard is measured. Due to coating, its mean *Z* value is reduced compared to the value of pure aluminum, and the gray-level histogram is shifted to values below 225. The thickness of the carbon layer on each sample is in the range of several tens of nanometers. Its exact value cannot be controlled. Thus, the gray-level reduction due to carbon coating varies from sample to sample. This effect is corrected using an aluminum sample standard coated simultaneously with the sample. This procedure shall ensure that sample and sample standard have similar carbon layer thicknesses. Then, a comparison of the (carbon coated) sample standard to the main standard consisting of pure, uncoated aluminum, allows to estimate the GL reduction due to coating. Reductions of up to 2-gray levels are tolerated. When the reduction is larger than 2, brightness and contrast are changed such that the main standard shows gray levels of 25 and 223 for carbon and aluminum, respectively. Typically carbon coating reduces the measurement signal from 1 to 5 GLs. Then the measurement of the sample is performed. During measurement, the stability of the extractor current (the main regulator of the probe current) is monitored. After the measurement, the main standard is measured again. Shifts in the gray-level histograms of $\pm 1$ are accepted. Whenever stability of gray levels or extractor current is not achieved, the measurement is discarded.

### Data Evaluation

8-Bit gray-scale images with pixel dimension $512 \times 512$ for the DSM 962 and $1024 \times 768$ for the SEM SUPRA 40 are collected. In the latter device, the pixel size is quadratic, while in the former, the pixel size is a factor 1.27 larger in scan direction than perpendicular to it. The rectangular pixel shape in the DSM 962 has no influence for the calculation of the BMDD as described below. The BMDD is a frequency distribution and does not contain any geometrical information. Of course, the pixel shape has to be considered when histomorphometric information like, e.g., trabecular thickness or cortical width are inferred from the image. In particular this is true for the depiction of the images themselves. In the current manuscript, all images obtained with the DSM 962 are scaled to a pixel dimension of $650 \times 512$. In the case of the DSM 962, several non-overlapping areas of $2.4 \times 1.9 \ \text {mm}^2$ of the sample surface are scanned, while the entire sample surface is scanned with the SEM SUPRA 40. In the latter case, this allows for stitching the single pictures together to give an image of the entire surface of the sample of arbitrary pixel dimensions. In this case, images are recorded with a $5\%$ overlap. The stitching is performed with an ImageJ plugin from Stephan Preibisch et al. [41].

The images are subsequently processed based on self-written macros in ImageJ (https://imagej.nih.gov/ij/). All gray levels lower than 25 corresponding to PMMA and soft tissue of the bone marrow space are thresholded and set to gray level 0. Then the images are filtered and cleaned to remove debris and dirt. From the cleaned images, GL histograms are produced. Based on Eq. 4, these histograms are transformed into BMDDs that give the amount of bone surface mineralized with a certain calcium content. The BMDDs are normalized to $100\%$ bone area. These curves are further processed as described elsewhere [29]. In particular, 5 main parameters describing the curve are evaluated: (i) CaMean indicates the mean calcium content found in the sample

1 $$\begin{aligned} \text {CaMean} = \frac{1}{100} \sum \limits _{i=0}^{255} c_{i} \times \text {BMDD}_i \end{aligned}$$

Here, BMDD$_i$ is the *i*-th entry of the histogram and $c_{i}=0.1733*i-4.3326$ denotes the corresponding calcium concentration in wt%. (ii) CaPeak indicates the most frequently measured calcium content, i.e., the location of the peak of the curve. To reduce effects due to noise, CaPeak is not defined as the location of the maximum of the curve, but it is measured as the middle point between the locations where the BMDD has reached $75\%$ of the maximum value on both sides. (iii) CaWidth indicates the heterogeneity of the mineralization and is measured as full width at half maximum of the curve. (iv) CaLow and (v) CaHigh indicate the percentage of bone material that is mineralized less than the 5th percentile and more than the 95th percentile of the reference BMDD curve, respectively.

## Results

To answer the question, if the BMDD outcomes of both types of SEMs can be directly compared, the following analyses were performed: (i) The linear relation between backscattered electron yield and mean atomic number was verified for the two SEMs used (On the Relation Between Backscattered Electron Yield and Mean Atomic Number). (ii) The identical surface of the same sample was measured repeatedly with the same device. The time span between the measurements was even up to several years. This gave insight into the long-term stability of the devices, reproducibility of the measurement itself as well as on the stability of the resin embedded bone sample over time (Device Stability Over Time). (iii) A comparison of BMDDs obtained with the same device at different magnifications was performed to apprehend how different magnifications affect the obtained images (Measurements with the Same Device at Different Magnifications). (iv) BMDDs from the identical sample but measured with two different SEM devices at the same spatial pixel resolution were compared. This could clarify the quantitative comparability of BMDD curves obtained from different devices. The results showed that although both devices lead to qualitatively similar results, quantitatively the results were not equal. In particular, this means that BMDDs measured with the SEM SUPRA 40 cannot be used directly to compare them with the reference BMDD curves for healthy bone obtained with the DSM 962 [39] (Comparison Between Different Devices). (v) Consequently, it is described how new reference BMDDs were obtained for the new device (Reference BMDD Curves).

### On the Relation Between Backscattered Electron Yield and Mean Atomic Number

The main assumption of the qBEI method is that the backscattered electron coefficient, i.e., the number of electrons reflected back from the sample divided by the number of impinging electrons on the sample, shows—for light elements—a linear dependence on atomic number *Z* [28]. While for pure elements, the atomic number is known, in compounds, there is no natural choice on how to calculate a mean Z [42]. In a previous work from Roschger et al. [32]. the following formula suggested by Lloyd [43] was used:

2 $$\begin{aligned} Z_{\text {mean}} = \frac{\sum _{i} N_{i} A_{i} Z_{i}}{\sum _{i} N_{i} A_{i}}. \end{aligned}$$

Here, the sum runs over all elements *i* present in the compound; $N_{i}$, $A_{i},$ and $Z_{i}$ are denoting the corresponding number per unit weight of the compound, atomic weight, and atomic number, respectively. Thus, a necessary test for the applicability of the qBEI method is to verify the linear dependence of backscattered electron yield on the mean atomic number of the compound evaluated with Eq. 2. For the DSM 962, the linearity of the backscattered coefficient was verified for carbon, aluminum, fluorapatite, and MgF$_2$ (all with certified micro analytical measured elemental composition data) in a previous study [32]. In the current work, the linearity of the backscattered signal for the DSM 962 and SEM SUPRA 40 is verified by calibrating the device with the calibration reference standards of pure carbon and pure aluminum and subsequent gray-level measurements of 5 different minerals with estimated composition by semi-quantitative EDX analysis limited to an accuracy in elemental concentration of about 1 wt%.

**Fig. 3:**
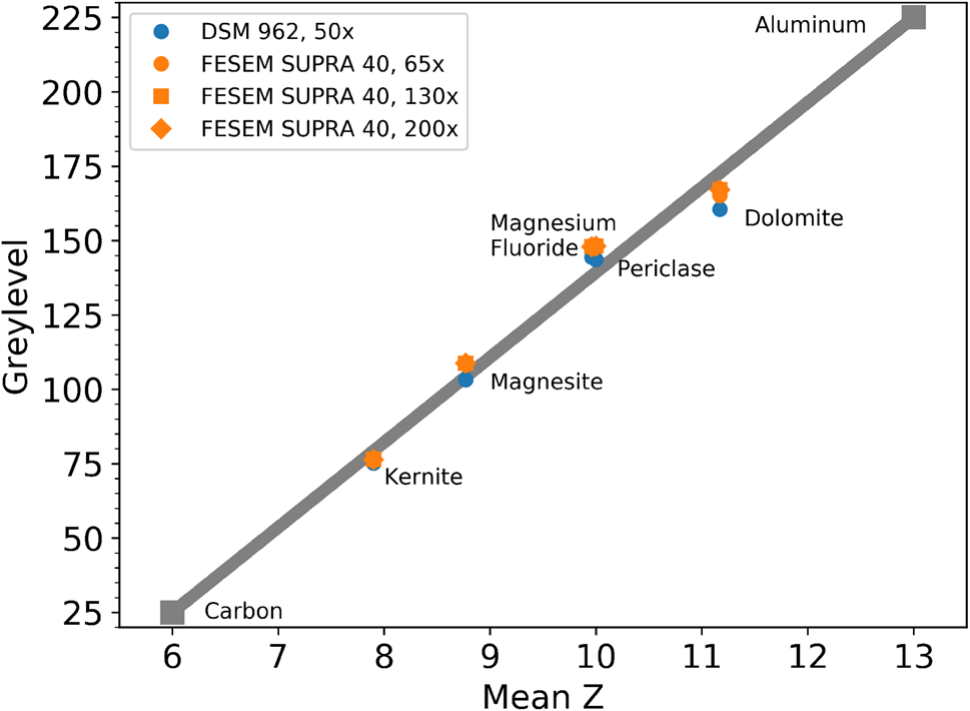
GLs measured for 5 different minerals: Kernite ($\text {Na}_2 [\text {B}_4\text {O}_6(\text {OH}_2)] \cdot 3\text {H}_2\text {O}$), Magnesite ($\text {MgCO}_3$), Magnesium Fluoride ($\text {MgF}_2$), Periclase (MgO), and Dolomite ($\text {CaMg}[\text {CO}_3]_2$) with two SEMs: a Zeiss DSM 962 equipped with a tungsten cathode and a Zeiss field emission SEM SUPRA 40. The mean *Z* number of the minerals was obtained by a compositional EDX analysis of the samples. GLs were measured with one nominal magnification of $\times$ 50 for the DSM962 and for three different nominal magnifications (× 65, × 130 and × 200) for the SEM SUPRA 40. The solid gray line denotes the calibration line: brightness and contrast of the detector are set such that pure carbon (*Z* = 6) and aluminum (*Z* = 13) have a gray level of 25 and 225, respectively. The thickness of the line corresponds to a $\pm 2 \ \text {GL}$ uncertainty due to instrument calibration and carbon coating

Figure 3 shows the obtained gray levels (GLs) for the minerals measured with the two different SEMs and different nominal magnifications. The solid gray line denotes the calibration line drawn between a GL of 25 for carbon ($Z_{C}=6$) and GL 225 for aluminum ($Z_\text{{Al}}=13$), respectively. The thickness of the line corresponds to the uncertainty of $\pm 2$ GL in the calibration routine and due to carbon coating. Three observations can be made: first, the measured gray levels fall approximately on the calibration curve for both devices. Second, there are only slight variations in gray level when the same mineral is measured with different devices. Third, the results do not depend on magnification (for low magnifications up to a × 200). These results allow to use the calibration line as a linear relation between gray level *GL* and mean atomic number $Z_{\text{mean}}$ as follows:

3 $$\begin{aligned} GL = 200 \left( \frac{Z_{\text {mean}}-Z_{\text {C}}}{Z_{\text {Al}}-Z_{\text {C}}} \right) +25. \end{aligned}$$

As shown previously, under the assumption that bone is a mixture of stoichiometric hydroxylapatite and collagen the mean atomic number of bone depends linearly on calcium content. Consequently, the measured GL can be translated into local wt% Ca via [31, 32]

4 $$\begin{aligned} \text {wt\% Ca} = k \times \text {GL} - d. \end{aligned}$$

The described calibration of the SEM device ensures that pure hydroxylapatite with 39.86 wt% Ca and $Z_{mean}=14.06$ yields a GL of 255, while carbon with zero wt% Ca and $Z_{\text {mean}}=6$ gives a GL of 25. Thus, it is found that

5 $$\begin{aligned} k= & {} \frac{39.86}{230} = 0.1733. \end{aligned}$$

6 $$\begin{aligned} d= & {} -25 \times k = -4.3326. \end{aligned}$$

### Device Stability Over Time

**Fig. 4:**
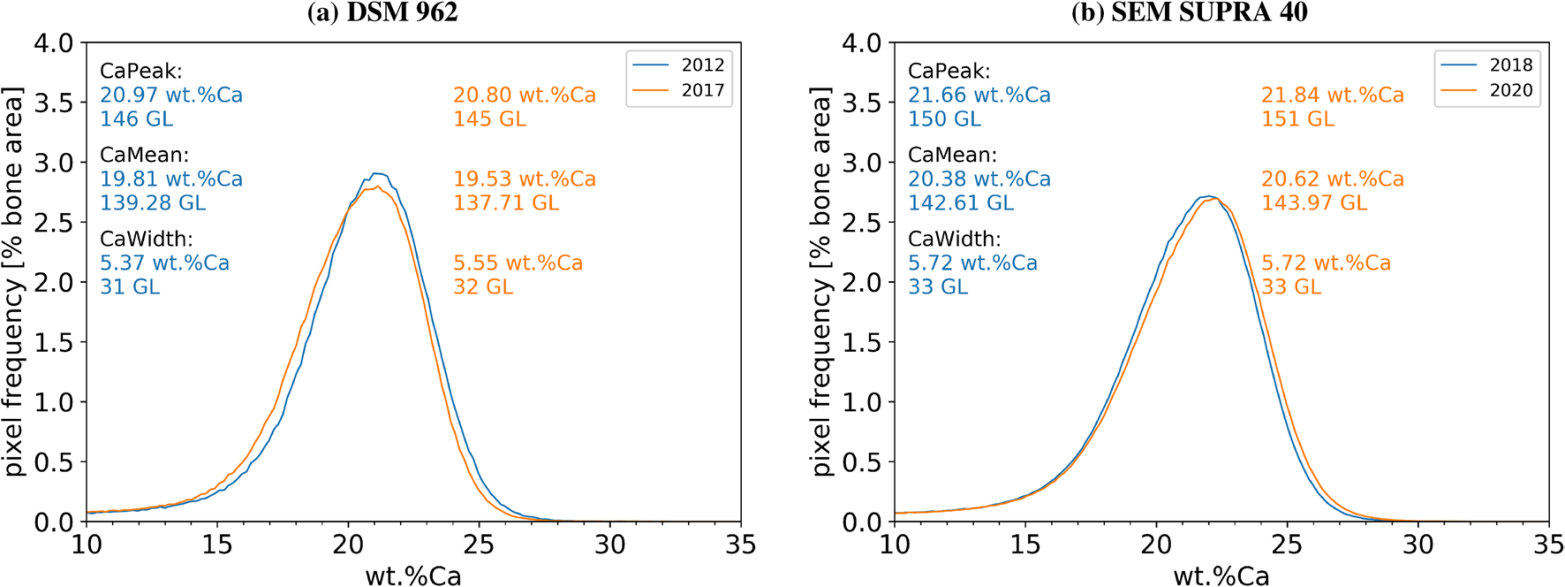
Repeated measurement of one sample on the same device. **a** BMDD from an iliac crest biopsy sample measured with the DSM 962 in the year 2012 and 2017, respectively. **b** The same sample measured on the SEM SUPRA 40 in 2018 and 2020, respectively. Additionally, CaPeak, CaMean, and CaWidth are given for the two curves (in wt% Ca as well as in GLs). The measured parameters do not differ more than 2 GLs

To test the (long term) stability of the devices and the reproducibility of measurements in time, iliac crest bone samples were measured repeatedly with the same device with a time lag of several years between the measurements. Figure 4 shows the results: (A) contrasts measurements from the same sample taken in 2012 and 2017 measured with the DSM 962. Obviously, the measured curves coincide almost perfectly. This is also confirmed by the values of the parameters describing the shape of the BMDD: CaPeak, CaMean, and CaWidth do not differ more than 2 GLs between the measurements. This is compatible with the $\pm 1$ GLs changes accepted for the reference standards before and after measurement. (B) A similar result of reproducibility is achieved for a sample measured repeatedly on the SEM SUPRA 40. Here, a measurement performed in 2018 was repeated 2 years later in 2020. Also for these measurements, the BMDD parameters do not differ more than 2 GLs. This remarkable reproducibility of the qBEI measurements also indicates that the temporal stability of the PMMA embedded bone sample is high.

### Measurements with the Same Device at Different Magnifications

**Fig. 5:**
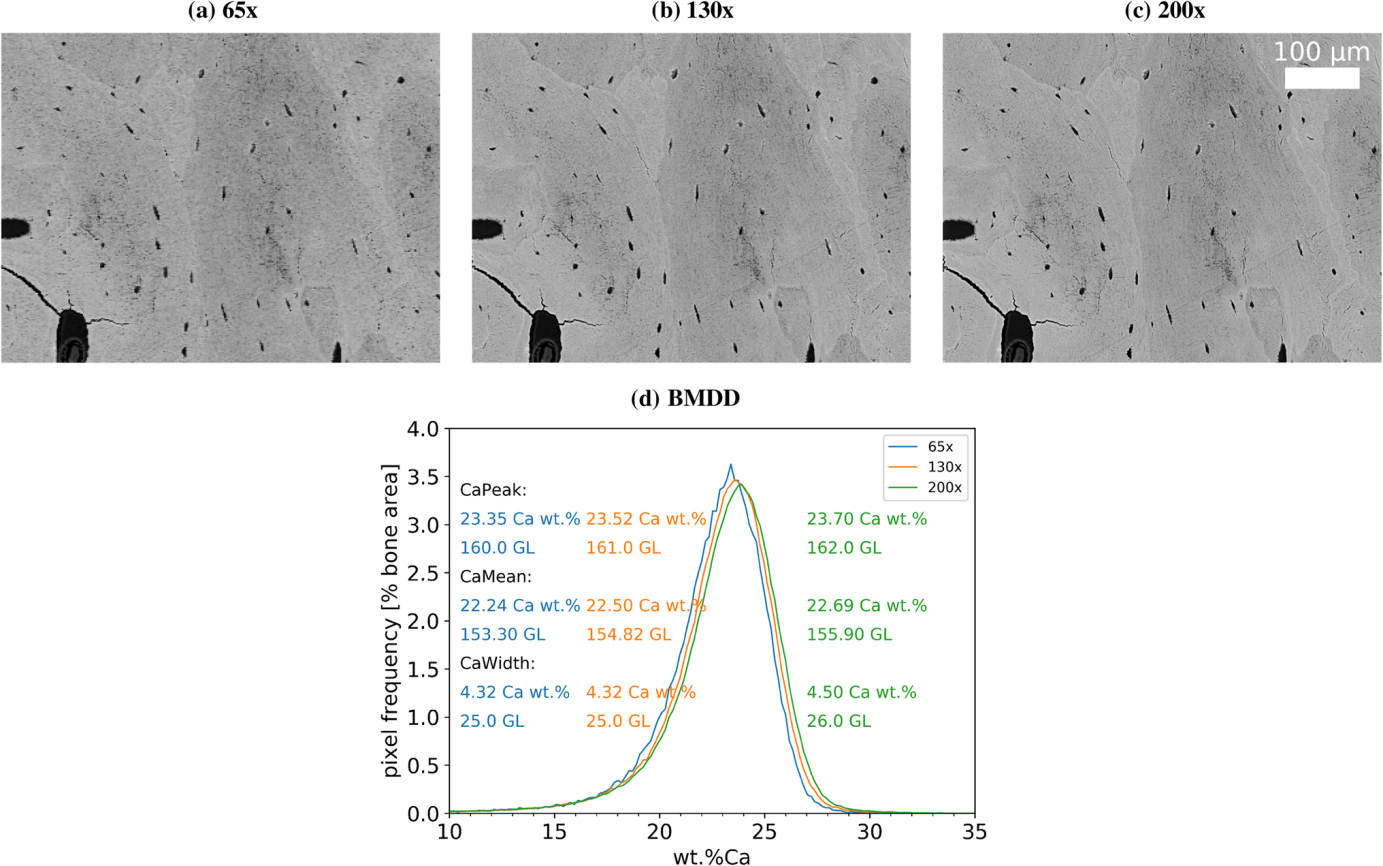
Typical images obtained from measurements of the same ROI with different nominal magnifications and corresponding BMDD curves. **a** × 65 with pixel resolution 1.76 μm, **b** × 130 with pixel resolution 0.88 μm, **c** × 200 with pixel resolution 0.57 μm. All images show the same area of $587 \times 440 \ \mu \text {m}^2$ and have be obtained with the SEM SUPRA 40. **d** Corresponding BMDDs

Another question concerns measurements on the same device with different magnifications. In Fig. 5, we show an example of images and BMDDs of one and the same bone surface measured with three different magnifications on the SEM SUPRA 40. It was ensured that the three measurements were performed on exactly the same bone area. As the pixel resolution changes with magnification, this means that the obtained images have different pixel dimensions for the three investigated magnifications: $333 \times 250$ (mag. $\times 65$), $665 \times 499$ (mag. $\times 130$) and $1024 \times 768$ (mag. $\times 200$). Hence, observed differences in the measurements can be solely attributed to the changed magnification. The results show that there is a small shift to higher GLs with increasing magnification (CaPeak and CaMean increase approximately 2 GLs from magnification × 65 to × 200). Furthermore, with increasing magnification also, a small broadening (increase in CaWidth) of the curve (one GL from magnification × 65 to × 200) can be observed. Of note, the extent of the shift to higher CaPeak and CaWidth values with increasing magnification varied considerably with different bone samples.

### Comparison Between Different Devices

**Fig. 6:**
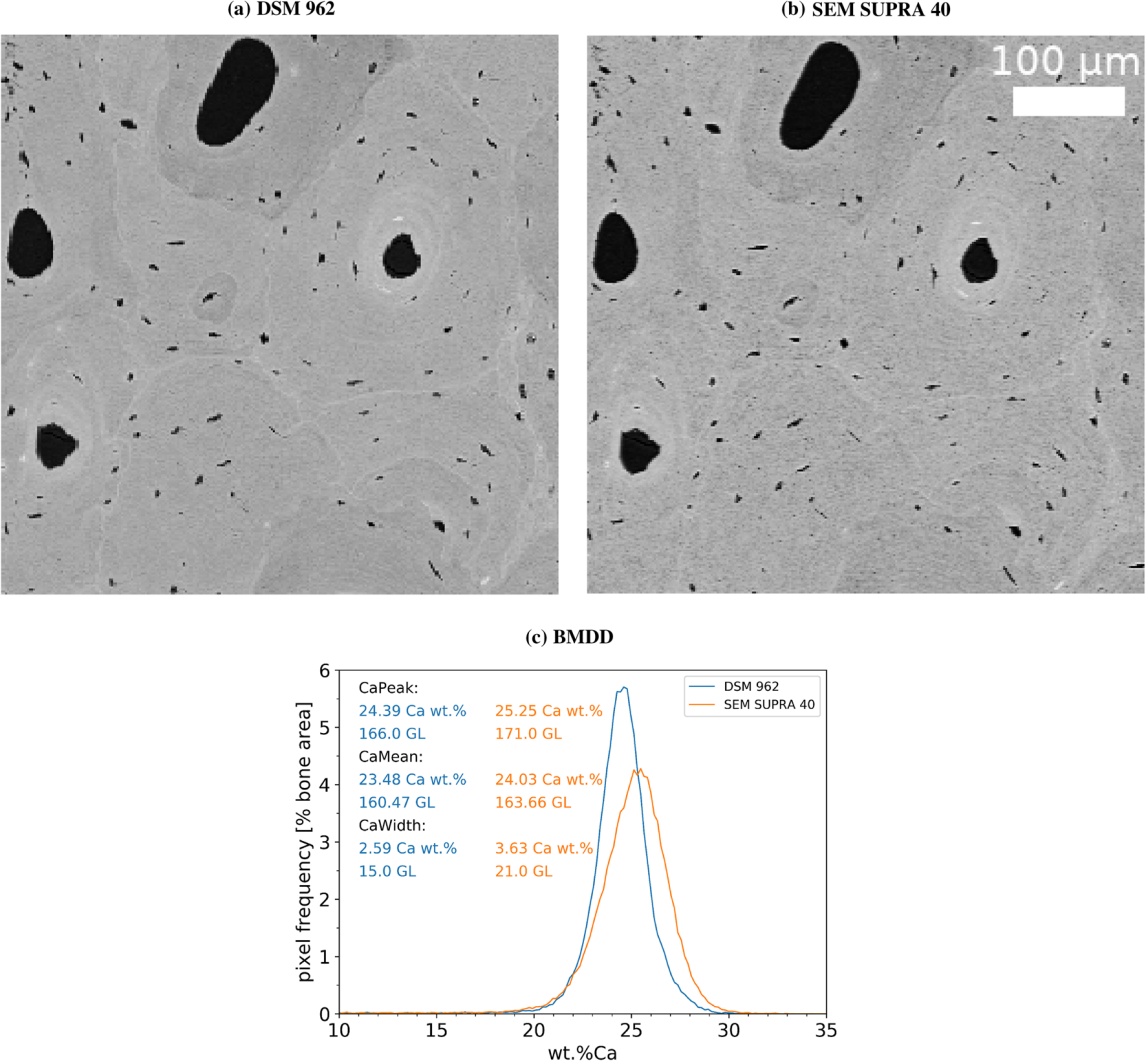
Images and corresponding BMDD curves of the same surface (covering a region of $500 \times 500 \ \mu \text {m}^2$) measured with the same resolution (1.76 μm/pixel) with both devices. **a** Image obtained with the DSM 962 and **b** with the SEM SUPRA 40. The same features (bone packet size and shape) can be observed in both images shown. **c** The BMDD curves show that there is a significant shift to higher mineralizations and broadening of the curve for the SEM SUPRA 40 compared to the DSM 962

Another point concerns the comparison of measurements obtained from the two different devices at very similar spatial resolution. Figure 6 shows a comparison of the same surface investigated with pixel resolution $2.24 \times 1.76 \ \mu \text {m}^2$ (DSM 962) and $1.76 \times 1.76 \ \mu \text {m}^2$ (SEM SUPRA 40). Note that the pixel resolution for the DSM 962 is rectangular and can, thus, only be matched with one dimension for the quadratic pixel size in the SEM SUPRA 40. The same features (size and shape of bone packets) can be qualitatively observed in both images. Nevertheless, the image obtained with the SEM SUPRA 40 appears to be structured on a smaller scale compared to that acquired with the DSM 962: (i) Qualitatively, this can be visualized by smearing of the cement lines in the SUPRA 40 image compared to the DSM 962 image. (ii) Quantitatively, it can be noticed in the BMDD analysis of the images showing a shift to higher mineralizations (for instance, CaMean was found to be increased up to 1 wt% Ca) and a broadening of the curve obtained from the SUPRA 40 compared to the DSM 962.

### Reference BMDD Curves

**Fig. 7:**
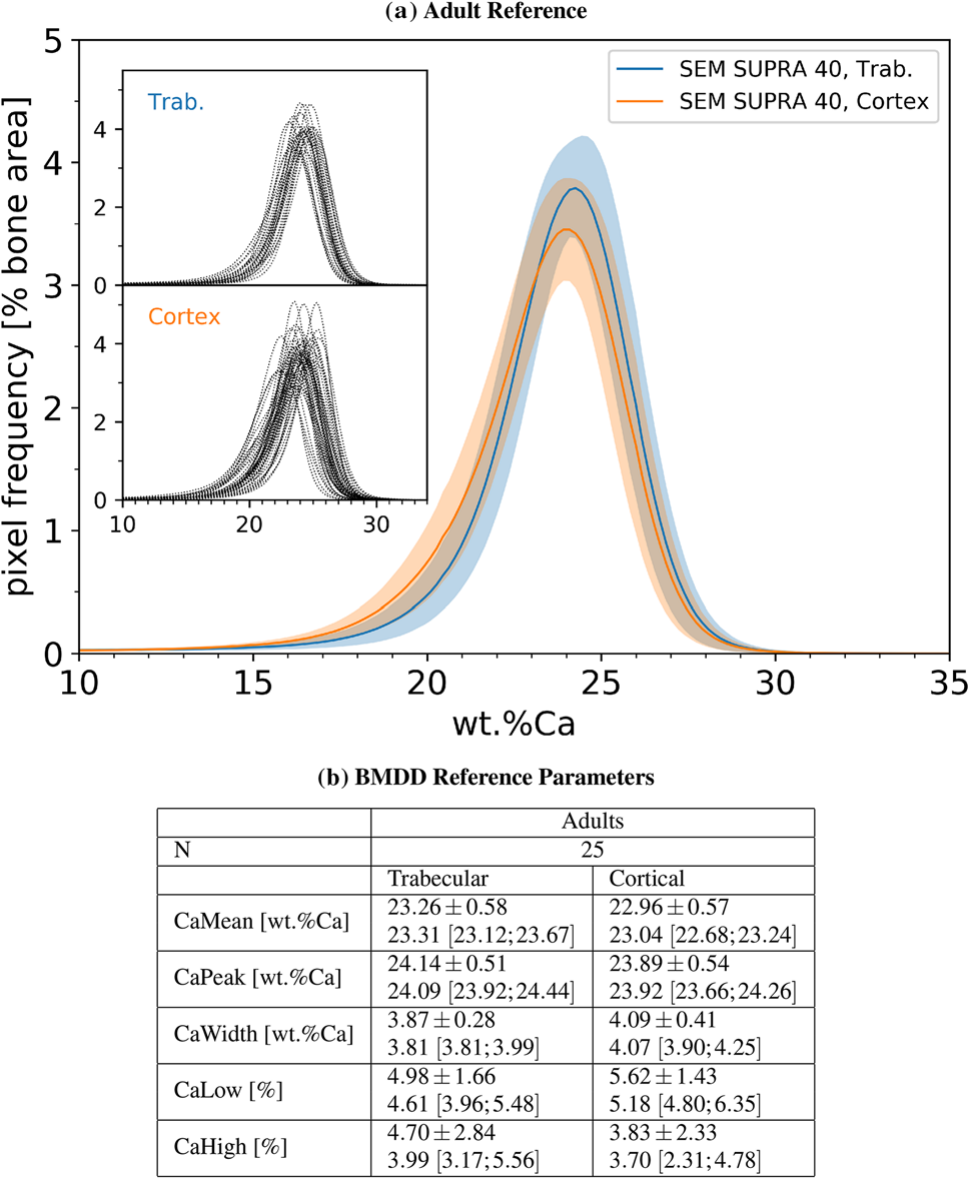
Adult trabecular and cortical references and corresponding BMDD parameters obtained from the SEM SUPRA 40. **a** Adult trabecular and cortical reference BMDDs. The insets show the single BMDDs used for averaging to obtain the corresponding reference BMDD. **b** The obtained BMDD parameters for the presented single BMDD curves are shown as mean ± SD and median [25%; 75%], respectively. Median and quartiles are obtained using the python numpy library

The previously reported trabecular reference BMDD for adults was obtained with the DSM 962 at a pixel resolution of $4.54 \times 3.57 \ \mu \text {m}^2$ [39]. This pixel size was found to be suitable to quantify the mineral content within the individual bone structural units (BSUs) that bone is composed of, while keeping the number of the scanned images low. In the SEM SUPRA 40, the pixel size compatible with the instrumental parameter settings for qBEI was found to be not larger than $1.76 \times 1.76 \ \mu \text {m}^2$. However, the new instrument is equipped with an automated scan procedure. This allows to record single images from the entire bone surface. Subsequently, the images can be stitched to obtain one large qBEI image of the whole bone area. Thus, the qBEI-BMDD analysis became even faster with the SEM SUPRA 40 compared to the DSM 962, although the former requires to acquire a larger number of single images. Moreover, the additional advantage of using a smaller pixel size is that it reduces the partial filled volume effect as discussed in [29]. As shown in figure 5, such a change in pixel size alone can induce changes in BMDD outcomes. Further, Fig. 6 shows that there is also a significant change in the BMDD to higher CaPeak and larger CaWidth values when switching to the SUPRA 40 device at the same pixel resolution. Hence, it is evident that BMDDs acquired with the SEM SUPRA 40 cannot be compared against the reference curves obtained with the DSM 962. Consequently, new references for the SEM SUPRA 40 have been recorded. The results are summarized in Fig. 7: (A) shows the trabecular and cortical reference BMDDs for adults obtained with the SEM SUPRA 40. The BMDD peak shows consistently a shift to higher mineralizations and a broadening compared to the previously reported BMDD obtained with the DSM 962 [39]. Moreover, the shifts are of the same order of magnitude as found for the single BMDDs shown, e.g., in Fig. 6. The trabecular and cortical reference BMDD obtained with the SEM SUPRA 40 are similar in peak position and shape. (B) shows that the BMDD parameters for cortical and trabecular bone all lie within one SD reflecting the similarity between trabecular and cortical BMDD of transiliac bone samples. New CaLow and CaHigh values are obtained as percentage of bone area mineralized below 18.20 wt% Ca and above 26.86 wt% Ca, respectively. These values correspond to the 5th and 95th percentiles of the new trabecular reference BMDD for adults.

## Discussion

The measurements of the backscattered electron yield as a function of mean atomic number *Z* of different mineral crystals confirmed a linear dependence in the range of *Z* corresponding to bone ($Z \approx 10$). This linear dependence was found equal for different pixel resolutions and both devices with different electron sources (DSM 962 with a tungsten hairpin cathode and SEM SUPRA 40 with a field emission cathode). These results justify the use of Eq. 4 to relate measured GL and local calcium content in bone samples that lies at the heart of the qBEI method for SEMs independent of their specific electron source.

Further, we observed that there is a clear broadening of the BMDD peak as well as a GL shift in the BMDDs to higher mineralization levels when measured at similar pixel resolution by SEM SUPRA 40 compared to DSM 962. Such a shift is consistently found for all samples measured and is in the range up to 1.0 wt% Ca for the BMDD parameter CaMean. One of the main reasons for the observed discrepancies seems to be the difference in beam characteristics for the two devices. The electron source size is 100 to 1000 times smaller for the field emission compared to the tungsten cathode. The smaller source size causes a much less divergent and smaller beam diameter at the imaged sample surface for the SEM SUPRA 40 compared to the DSM 962 [28]. One particular feature of a field emission microscope is that the beam size is the same for all nominal magnifications, while for the DSM 962 the beam size has to be adjusted for each magnification. Consequently, the beam electron interaction volume in the sample material, respectively, the escape area of the backscattered electrons from the sample surface, is much smaller for the SEM SUPRA 40 compared to the DSM 962 (nevertheless, still much larger than the beam diameter for both devices [28]). Most importantly, for both devices, the escape area is smaller than the nominal magnification given by the pixel size. This means that not everything located in the pixel contributes to the measured signal. Therefore, some details of the structure (such as cement lines) may actually be under-sampled or even missed in the SEM SUPRA 40 mapping. This explains the observation of smearing of cement lines in the SEM SUPRA 40 compared to the DSM 962 presented in Fig. 6. A measurement with higher pixel resolution will, however, reveal many more details in the device with the field emission cathode (see, e.g., the gradual increase in magnification for the SEM SUPRA 40 in Fig. 5). The consequence is that the lower inherent resolution of the DSM has some advantages for (low resolution) qBEI mapping, while the field emission microscope has clear advantages for high-resolution imaging. This effect is likely leading to the described alterations in BMDD characteristics compared to the DSM 962. Noteworthy that in case of homogeneous samples like *Z* standards, differences in beam diameter at focal plane also lead to consistently higher BE signal outcomes for the SEM SUPRA 40 compared to the DSM 962. In contrast, differences in magnification do not change the BE signal outcomes for the SEM SUPRA 40, which are consistent with the fact that in this device, spot size (beam diameter at sample surface) does not change with magnification (see Fig. 3). Both observations support the assumption that the above-described effect of BMDD GL shift originates from the inhomogeneous hierarchically structured bone material. Consequently, a numerical rescaling of the BMDD curves of the FE-SEM is not feasible as the shift in GL between the two devices is not a function of the device alone but depends also on the ultrastructural characteristics of the sectioned bone sample surface.

In contrast, measurements with the identical device are highly reproducible over the range of years with a variation of $\pm 1$ GL, i.e., 0.17 wt% Ca. This is much better than the variations of up to $\pm 6$ GL (1.0 wt% Ca) found between the different devices. Consequently, the stability of the device (sample chamber, detector, and electronics) and also of the PMMA embedded sample itself, allows to compare measurements obtained from the same device over years.

These observations allow drawing the following conclusions: qBEI measurements can be quantitatively compared when they are (i) measured with the same device and (ii) measured under exactly the same conditions (especially the same magnification). If the stability of the device can be ensured the achieved relative accuracy (within one device) can be as good as 0.2 wt% Ca or $\pm 1 \ \text {GL}$. On the other hand, the different types of electron beams used in the two devices lead to differences in measured parameters of up to 1.0 wt% Ca or $\pm 6 \ \text {GL}$ for instance in case of CaMean.

In particular, this has implications on the trabecular reference BMDD for healthy adults published in reference [39]. In this paper, it was shown that the trabecular BMDD in healthy adults is independent of age, sex, skeletal site, and ethnicity. Consequently, measurements on arbitrary samples can be compared against these references to gain insight into the mineralization pattern, e.g., if the sample is normal-, hypo- or hyper-mineralized. As changes in BMDD parameters due to diseases, like e.g., osteoporosis or osteogenesis imperfecta, are often in the range of $\pm 1 \ \text {wt\% Ca}$ [29] which is similar to the changes due to different electron sources, direct comparisons between measurements obtained from different devices or obtained under different settings, e.g., magnification, are impossible. As the published reference was obtained with the DSM 962, they cannot be compared against BMDDs obtained with the newer device SEM SUPRA 40. Consequently, new reference curves for the SEM SUPRA 40 had to be obtained. Additionally to the trabecular reference BMDD, also a cortical reference was established from the same individuals at the same anatomical site (iliac crest).

## Conclusions

This paper discussed the dependence of the BE signal on the type of electron source present in the used SEM (thermionic or field emission cathode). Understanding this effect is of special importance when the BE yield is used to deduce information on the quantitative composition of a sample as is, e.g., done in a qBEI measurement on bone samples. It was shown that the linear relation of BE signal on sample composition is fulfilled for both investigated cathodes. Nevertheless, in addition to the atomic number contrast, a small gray-level shift to higher values was found for measurements with smaller electron beams obtained with a field emission cathode compared to a tungsten hairpin cathode. This shift was found consistently for pure and homogeneous *Z* standards as well as for hierarchical and inhomogeneous bone samples. The shift is small in absolute values, but in the same order of magnitude as changes in BMDD values due to diseases, like osteoporosis or osteogenesis imperfecta. On the other hand, measurements with the same type of cathode were found to be highly reproducible over time scales of several years. Nevertheless, on the same device, systematic shifts in BE yield were also detected for measurements with different magnifications. These results show that quantitative comparisons between qBEI measurements are only valid, when the measurements were obtained with the same device and the same measurement conditions. In particular, this has implications when BMDD curves are compared against reference values. The measured BMDD data can only be compared with data obtained with a similar device.
